# Profile and outcome of patients with Warfarin Toxicity admitted in a tertiary care hospital in Bhutan: a cross-sectional study investigators and institutions

**DOI:** 10.1186/s13104-023-06359-2

**Published:** 2023-05-18

**Authors:** Yeshey Penjore, Thinley Dorji, Sangay Dorji, Saran Tenzin Tamang

**Affiliations:** 1 Department of Internal Medicine, Jigme Dorji Wangchuck National Referral Hospital, Thimphu, Bhutan; 2 Department of Internal Medicine, Central Regional Referral Hospital, Gelegphu, 31101 Bhutan; 3 Faculty of Postgraduate Medicine, Khesar Gyalpo University of Medical Sciences of Bhutan, Thimphu, Bhutan

**Keywords:** Adverse drug event, Patient safety, Anticoagulants, Patient education

## Abstract

**Objective:**

To study the profile, clinical presentation and outcome of hospital stay among patients admitted with warfarin toxicity at the Jigme Dorji Wangchuck National Referral Hospital, Bhutan. This was a cross-sectional study with a review of hospital records of patients admitted between 01 and 2018 and 30 June 2020.

**Results:**

There were 22 admissions due to warfarin toxicity. The mean age of patients was 55.9 (± SD 20.2) years, the median duration of warfarin therapy was 30 months (IQR 4.8, 69 months). The indications for warfarin were atrial fibrillation (9, 40.9%), mechanical heart valves (6, 27.3%), deep vein thrombosis (6, 27.3%) and pulmonary thromboembolism (1, 4.5%). The mean of dosage of warfarin was 4.3 (± 2.6) mg and the cumulative dosage in the week prior to admission was 30.9 (± 18.6) mg. The mean of INR at presentation was 7.7 (± 4.3) with the maximum noted at 20. The patients presented with gastrointestinal bleeding, muscle haematomas, epistaxis and oral cavity bleeding. There was no mortality related to warfarin toxicity. The reasons for warfarin toxicity included patient dosing error and drug interactions. Warfarin therapy requires appropriate patient education, adequate facilities for follow-up and avoidance of warfarin wherever possible in clinical settings.

**Supplementary Information:**

The online version contains supplementary material available at 10.1186/s13104-023-06359-2.

## Introduction

Anticoagulants are reported as the most common cause of adverse events leading to emergency department visits [1]. Anticoagulants contributed the highest proportion of emergency visits (17.6%) and almost half of them (48.8%) required hospital admissions in a review of 42,585 cases of adverse drug events reported in the United States [1]. Amongst anticoagulants, warfarin was implicated in an 85.7% of emergency visits among those aged older than 65 years compared to target-specific oral anticoagulants (apixaban, dabigatran and rivaroxaban) [1].

The common side effects of warfarin are haemorrhage (mucocutaneous, central nervous system, haemothorax), skin necrosis, vasculitis, agranulocytosis, albuminuria, calciphylaxis, diarrhoea, renal tubular acidosis and teratogenicity [2]. Warfarin has a narrow therapeutic index and its therapeutic dosage is monitored with International Normalized Ratio (INR) [2, 3]. There is a direct relationship between increased INR and higher risk of bleeding [4]. The risk of adverse events are higher in patients with comorbidities such as chronic kidney disease, liver disease and those on chemotherapeutic agents [5].

In addition to the sociodemographic (patient’s age, weight, body mass index, sex, smoking status, existing conditions, and concomitant medications) and compliance factors, there are well-characterized genetic polymorphism of CYP2C9 and VKORC1 gene that influence the rate of warfarin metabolism [3]. In persons with CYP2C9*3-homozygous individuals, the rate of warfarin clearance is about 10% of normal values and they have a much lower tolerance for the drug and have high risk for adverse effects. VKORC1 gene polymorphism is responsible for expression of vitamin K epoxide reductase and therefore determines the maintenance doses of warfarin. However, access to such testing is limited by cost and availability in many low- and middle-income countries.

**Fig. 1:**
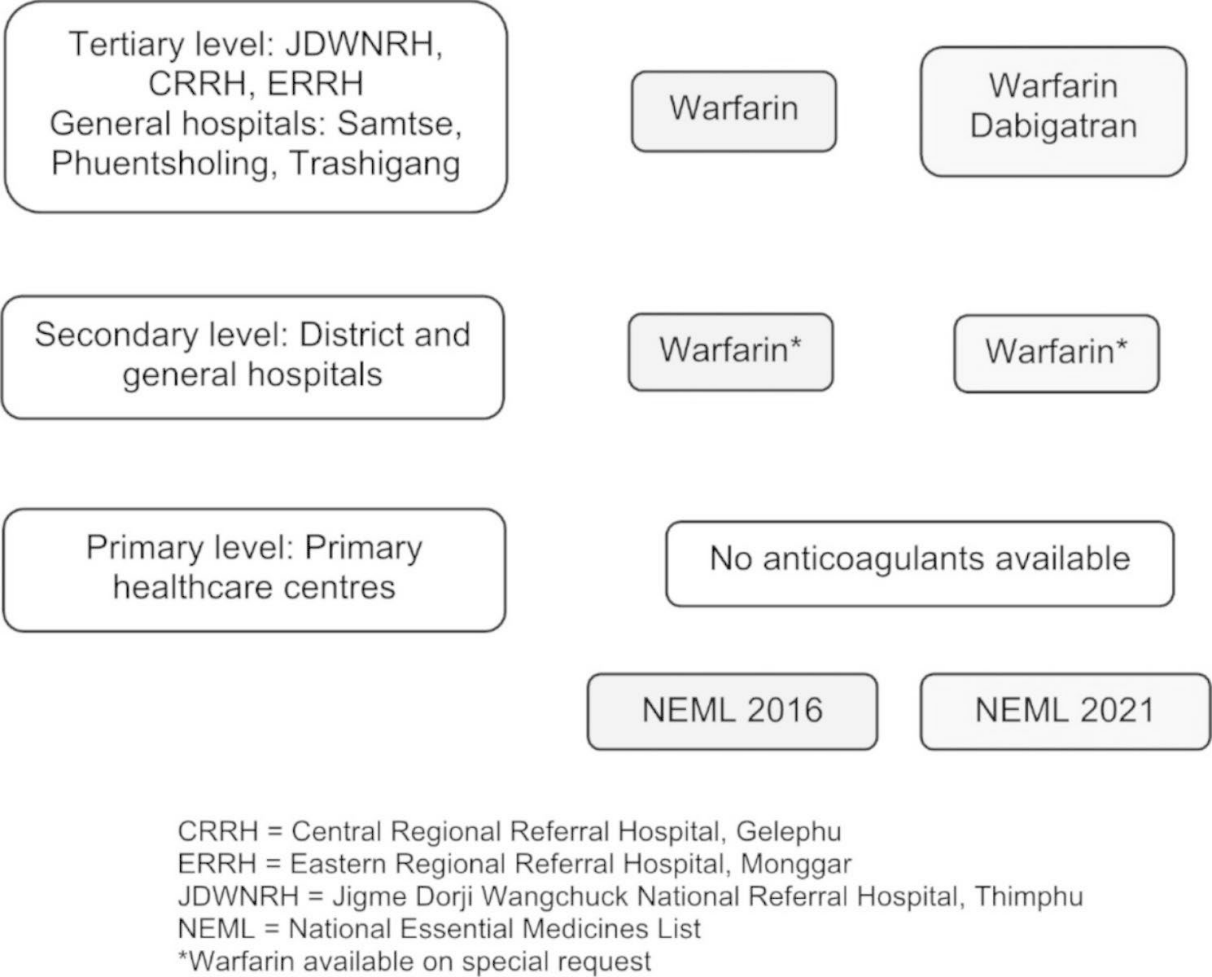
Levels of hospitals in Bhutan and the availability of anticoagulants through the National Essential Medicines List

In Bhutan, healthcare is provided free of cost under the National Health Policy 2011 and the Constitution of Bhutan through a three-tiered system [6]. The primary health centres and the 10-bedded hospitals are at the primary level, district and general hospitals are at the secondary level and the National Referral Hospital and the Regional Referral Hospitals are at the tertiary level [6]. Supply of medicines to the three levels of hospitals in the country is guided by the National Essential Medicines List 2021 [7]. Warfarin and Enoxaparin are available only in the three tertiary hospitals (national and regional referral hospitals) as shown in Fig. 1 [7]. Other anticoagulants are available to select patients through a special procurement process. Warfarin therefore is a common choice for prescription. Many patients lack timely and adequate INR monitoring and warfarin is often under- or over-dosed leading to many clinical complications and hospitalizations. However, the burden of hospitalization due to warfarin overdosage is not yet studied in Bhutan. This study describes the profile of patients admitted with warfarin toxicity at the Jigme Dorji Wangchuck National Referral Hospital from July 2018 to June 2020.

## Main text

### Methods

#### Study design

This was a cross-sectional study with review of hospital records of patients admitted with warfarin toxicity at the Jigme Dorji Wangchuck National Referral Hospital, Thimphu from 01 July 2018–30 June 2020.

### Setting

This study was conducted at the Jigme Dorji Wangchuck National Referral Hospital, Thimphu, Bhutan. It is the largest hospital in the country catering to 605,398 of out-patient visits in 2021 [8]. Patients with warfarin toxicity present to the outpatient clinics and the emergency department while a proportion of the patients are referred from district or other referral hospitals. The records of these patients are stored in the Medical Records Section of the hospital. The case files are coded with ICD-10 coding.

### Study population and sample size

In 2017, 1644 patients were admitted in Medicine Wards [9]. The proportion of admissions due to warfarin toxicity is not known. In our study, data of all patients who were admitted with warfarin toxicity were included. Those with incomplete set of data regarding warfarin use were excluded.

### Data variables, sources and data collection method

The data were extracted from the patient files stored at the Medical Records Section of the hospital and were recorded on a paper-based pro forma that was designed for the purpose of this study (Supplementary file 1). This included clinical note, investigation and laboratory reports. Search for patient records were summoned with the ICD-10 code T45.511 A for accidental or intentional poisoning by anticoagulants in the hospital’s data base. Other search words such as “warfarin toxicity” and “warfarin overdose” were used.

We also searched the database with the following ICD-10 codes: I85.0 for oesophageal varices with bleeding, K25 for gastric ulcer with bleeding, K26 for duodenal ulcer with bleeding, K27 for peptic ulcer with bleeding, K92.1 for malena, K60 for subarachnoid haemorrhage, K61 for intracerebral haemorrhage, K62 for subdural haemorrhage, R04.0 for epistaxis, R04.2 for haemoptysis, N02 for haematuria, H35.6, H43.1 and H45 for bleeding into the optic globe, R58 for haemorrhage not specified. These files were retrieved and assessed if patients were on warfarin.

### Data analysis and statistics

Data were double entered and validated using EpiData (version 3.1) and analysed using STATA (version 13.1, StataCorp LP USA).

Continuous variables are summarized using means, standard deviations, median and interquartile range. Categorical variables are summarized using frequencies, proportions and percentages. The severity of bleeding at presentation were categorized into four classes based on International Society of Thrombosis and Haemostasis (ISTH) recommendations [10]. The bleeding episodes were attributed to warfarin toxicity based on clinical evaluation and as documented by the clinicians.

### Ethics considerations

This study was performed in accordance with the Declaration of Helsinki and was approved by the Research Ethics Board of Health, Ministry of Health, Bhutan (approval no REBH/Approval/2020/059 dated 28/09/2020). The need for informed consent was waived by the Research Ethics Board of Health, Ministry of Health, Thimphu, Bhutan because this was a review of hospital records and not patient identifiers were collected.

## Results

There were 22 patients admitted with clinical manifestations of warfarin toxicity. The mean age of the sample was 55.9 (± SD 20.2) years and the majority were aged ≥ 55 years. There were 13 females (59.1%), the majority could not read and write (18, 81.8%). The details of the sociodemographic profile of patients are shown in Table 1.

**Table 1:** Sociodemographic profile of patients who were admitted due to warfarin toxicity at the Jigme Dorji Wangchuck National Referral Hospital, Thimphu between 2018–2020

Sociodemographic profile	n	(%)
Total	22	(100)
Age		
25–34 years	4	(18.2)
35–44 years	3	(13.6)
45–54 years	2	(9.1)
55–64 years	6	(27.3)
≥ 65 years	7	(31.8)
Sex		
Male	9	(40.9)
Female	13	(59.1)
Residence		
Rural	9	(40.9)
Urban	13	(59.1)
Level of education		
Cannot read and write	18	(81.8)
Monastic education / Non-Formal Education*	2	(9.1)
Class PP – VIII	1	(4.5)
Class IX – XII	0	(0.0)
Diploma / Undergraduate / Masters	1	(4.5)

*Monastic education refers to Buddhist education in Dzongkha language relating mostly to religious study. Non-Formal Education refers basic literacy education provided to adults who have missed opportunities for formal school education

**Fig. 2:**
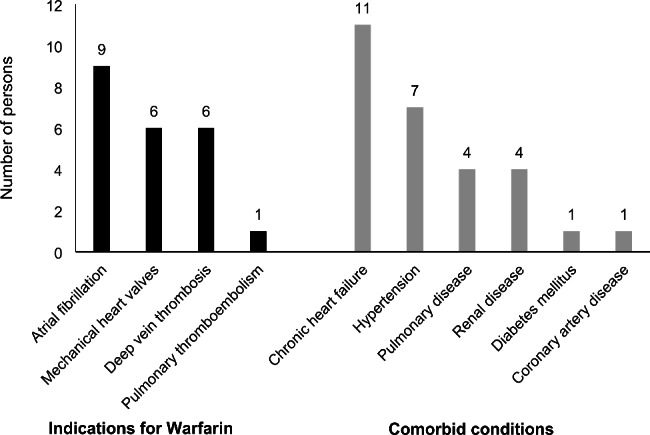
Indications for warfarin and co-morbid conditions among patients who were admitted due to warfarin toxicity at the Jigme Dorji Wangchuck National Referral Hospital, Thimphu between 2018–2020

The indications of warfarin therapy included atrial fibrillation (9, 40.9%), mechanical heart valves (6, 27.3%), deep vein thrombosis (6, 27.3%) and pulmonary thromboembolism (1, 4.5%). The co-morbid conditions included chronic heart failure, hypertension, pulmonary hypertension, renal disease and diabetes (Fig. 2). The concomitant medications at the time of hospital warfarin toxicity included antiarrhythmics, antibiotics, antihypertensives, atorvastatin and high dose aspirin. All the patients administered the warfarin dosage by themselves. The median duration for which patients had been taking warfarin was 30 months (IQR 4.8, 69 months). Three patients (13.6%) had history of recent hospital admission with bleeding manifestations. The details of the clinical profile of patients are shown in Table 2.

**Table 2:** Clinical profile, presentations and outcomes of patients who were admitted due to warfarin toxicity at the Jigme Dorji Wangchuck National Referral Hospital, Thimphu between 2018–2020

Variables	n	(%)
Total	22	(100)
**Clinical profile**		
Warfarin dose administered by self	22	(100.0)
Recent hospital admission with bleeding	3	(13.6)
Concomitant drug		
Antiarrhythmic*	13	(59.1)
Antibiotics**	10	(45.5)
Atorvastatin	2	(9.1)
Low dose aspirin (< 100 mg)	2	(9.1)
High dose aspirin (≥ 100 mg)	0	(0.0)
Presumed cause of warfarin toxicity		
Dosing error by patient	12	(54.5)
Drug interactions	3	(13.6)
Drug interaction – unknown agent	1	(4.5)
Cause not identified	6	(27.3)
**Clinical presentation and outcomes**		
Clinical presentations		
Bleeding	19	(86.4)
Skin necrosis	3	(13.6)
Admission from		
Emergency Department	19	(86.4)
Outpatient department	2	(9.1)
Referral	1	(4.5)
Treatments provided		
Stopped Warfarin	19	(86.4)
Reduced dose of Warfarin	10	(45.5)
Vitamin K	15	(68.2)
Fresh frozen plasma transfusion	20	(90.9)
Packed red blood cell transfusion	15	(68.2)
Outcomes		
Discharged	22	(100)

*Antiarrhythmic: amiodarone

**Antibiotics: Amoxicillin, Ciprofloxacin, Doxycycline

On clinical evaluation, the presumed cause for warfarin overdose was patient error in dosing in 12 patients (54.5%) where patient had consumed higher dosage than that prescribed, drug interactions in three (13.6%), drug interaction with unknown alternative substance in one (4.5%) and the cause could not be identified in six patients (27.3%). Those with gastrointestinal bleeding and muscle hematomas had presented with ISTH severity grading 4.

The most common presentation to hospital was bleeding manifestation in 19 patients (86.4%) as shown in Table 2. The bleeding manifestations included gastrointestinal bleeding, soft tissue haematomas, oral cavity bleeding, epistaxis and hemarthrosis. The sites of bleeding and the ISTH severity grading are shown in Table 3.

**Table 3:** Bleeding manifestations and severity among patients who were admitted due to warfarin toxicity at the Jigme Dorji Wangchuck National Referral Hospital, Thimphu between 2018–2020

Bleeding manifestations	ISTH* 0	ISTH 1	ISTH 2	ISTH 3	ISTH 4
Epistaxis	-	-	-	2	-
Oral cavity	-	-	3	1	-
Gastrointestinal bleeding	-	-	1	-	6
Haematuria	-	-	-	-	1
Muscle hematomas	-	1	-	-	5
Hemarthrosis	-	-	-	-	1
Central nervous system bleeding	-	-	-	-	-
Other bleedings**	1	-	-	-	4

*ISTH = International Society of Thrombosis and Haemostasis [10]

**Other bleedings: haematuria, haemoptysis, heavy menstrual bleeding (02 cases), haemoperitoneum

The mean of dosage of warfarin was 4.3 (± 2.6) mg and the cumulative dosage in the week prior to admission was 30.9 (± 18.6) mg. The mean of INR at presentation was 7.7 (± 4.3) with the maximum noted at 20.

The treatment practices included stopping warfarin in 19 patients (86.4%), transfusion of fresh frozen plasma in 20 patients (90.9%), administration of vitamin K in 15 patients (68.2%) and transfusion of packed red blood cells in 15 patients (68.2%). There was no mortality related to warfarin toxicity.

## Discussion

The use of warfarin for anticoagulation is on the decline with the advantages of easy use, similar efficacy and the need for not requiring intensive monitoring. However, many low- and middle-income countries continue to use warfarin because of the easy availability and low cost. Bhutan is one such country where prescribers are allowed to dispense medications only from the National Essential Medicines List. Until its revision in 2021, the National Essential Medicines List allowed only warfarin as an anticoagulant in the country [11]. While many problems of supra- and sub-therapeutic anticoagulation were observed in clinical settings across the country, a proper analysis and clinical data is deficient given the lack of proper reporting and review in the country. This is the first study that reports the clinical scenario surrounding hospital admissions resulting from warfarin toxicity. Between 2018 and 2020, there were 22 cases that required hospital admission at the Jigme Dorji Wangchuck National Referral Hospital. The majority of the patients were aged ≥ 55 years and could not read and write. Among 235 patients on warfarin in a clinic in India, the majority who had bleeding were in the age group 41–61 years [12]. In conditions where poly pharmacy is common, non-vitamin K oral anticoagulants will be beneficial in terms of patient safety.

The common indications of warfarin therapy included atrial fibrillation, mechanical heart valves, deep vein thrombosis and pulmonary thromboembolism. The efficacy and safety benefits of non-vitamin K oral anticoagulants (NOACs) is known in these conditions [13]. The emerging indications where NOACs outperform warfarin are in cancer-associated thrombosis and cortical venous thrombosis. Warfarin performs better in patients with mechanical prosthetic valve replacements and in those with antiphospholipid syndrome [13, 14]. These indications need to be viewed in the background of disease epidemiology in the context of Bhutan where rheumatic heart diseases and valve replacements are common.

One major drawback of warfarin therapy is the need for INR monitoring. As of 2021, INR monitoring is available to only select hospitals and leaving out patients in the majority of districts having to travel to a higher centre every month for INR testing and titration of warfarin dosage. This results in many cases of under- and over-dosing of warfarin with overdosing causing repeat hospital admissions in as much as 14%. On the other hand, the only NOAC approved under the National Essential Medicines List 2021 is Dabigatran and is available only at the three referral hospitals in Thimphu, Gelegphu and Monggar [7]. Also, Dabigatran requires overlap with parenteral anticoagulant at the time of its initiation; the parenteral forms of anticoagulants are also only available in the referral hospitals. Therefore, the access to NOACs is limited to only three referral hospitals in Thimphu, Gelegphu and Monggar. While the national essential medicines list is aimed at keeping the cost of medicines low for financing a free healthcare system, a careful cost-benefit analysis of warfarin vs. NOACs must be strongly considered in the scenario of Bhutan.

In our study, one of the major contributors of warfarin toxicity was medication errors. The general literacy among adults (15 years and older) in Bhutan stands at 66.6% while literacy among elderly population might be still lower [15]. In many low- and middle-income countries, the overall health literacy is low and given the many food and drug interactions of warfarin, an inappropriate dosing of the drug results in preventable hospital admissions and deaths. For example, a study in Nepal among patients with thromboembolic complications, the knowledge on warfarin use was poor with more than half of them unable to identify correct responses for critical patient-related literacy factors such as drug dosing, drug and food interactions [16]. In addition, like in many countries in the region, co-medication with herbal and traditional drug formulations are common and the potential risk of bleeding due to drug interaction is often overlooked by both allopathic doctors and traditional practitioners [17].

The majority of warfarin toxicity presented with bleeding manifestations with a severity of ISTH 4 in gastrointestinal bleeding and muscle hematomas. They were managed with oral vitamin K and fresh frozen plasma transfusion; prothrombin complex concentrates are not available in the country. Similar to that reported from a clinic in India, there were no cases of cerebral haemorrhage [12]. Bleeding manifestations among patients were reported at a mean warfarin dosage of 4.3 mg and a mean INR of 7.7 which indicates the need to consider food and drug interactions and the genetic polymorphisms. Among 295 patients on Warfarin in a clinic in Pakistan, patients had overall lower to moderate score on health-related quality of life assessment [18]. Therefore, it is recommended to use scoring systems such as SAMe-TT2R2 (*S*ex, *A*ge, *Me*dical history, *T*reatment, *T*obacco use, *R*ace) and ACAChE (*A*ntiplatelet use, *C*hronic kidney disease, *A*ge, *C*ongestive *h*eart failure, Left ventricular *E*jection fraction) scores to identify candidates who will perform poorly with warfarin [19, 20].

With increasing proportion of elderly population across low- and middle-income countries, the proportion of patients requiring anticoagulants are on the rise [21]. The findings from this study demonstrates that subsidization of costs of healthcare in relation to the choices of anticoagulants needs proper evaluation. While warfarin is cheaper, follow-up of patients is cumbersome and is associated with additional costs in the treatment of complications. We recommend a proper cost-benefit analysis and health equity parameters in terms of the choices of anticoagulants prescribed in a public-funded free healthcare system in Bhutan.

### Limitations

Patients presenting at emergency department with warfarin induced hemorrhages are usually admitted into Medicine Wards for further evaluation and management. This study may not capture mortality that may happen at homes or the non-severe cases that may be resuscitated and discharged from the emergency department. However, the proportion of both may be minimal. In the evaluation of causality, the relation to warfarin toxicity was based on clinician’s evaluation. No causality assessment scales could be applied because this was a retrospective review of data based on patient files.

## Conclusions

Warfarin toxicity is common among with older population and those who cannot read and write. One-tenth had history of multiple hospital admissions. Warfarin is prescribed for conditions where NOACs are recommended. This calls for a careful evaluation of cost-benefit analysis in the context of sustainable financing of the free healthcare system in Bhutan.

## Electronic supplementary material

Below is the link to the electronic supplementary material.

Supplementary Material 1

Supplementary Material 2

## Data Availability

The datasets generated and/or analysed during the current study is attached as supplementary file (Microsoft Excel).

## Abbreviations

ACAChE: *A*ntiplatelet use *C*hronic kidney disease *A*ge, *C*ongestive *h*eart failure, Left ventricular *E*jection fraction
INR: International normalised ratio
IQR: Inter-quartile range
ISTH: International Society of Thrombosis and Haemostasis
NOAC: Non-vitamin K oral anticoagulant
SAMe-TT2R2: Sex, Age, Medical history, Treatment, Tobacco use, Race
SD: Standard deviation
