# Supplementary material for: Profile and outcome of patients with Warfarin Toxicity admitted in a tertiary care hospital in Bhutan: a cross-sectional study investigators and institutions
Source: BMC Res Notes. 2023 May 18;16:81. doi: 10.1186/s13104-023-06359-2 (PMC10193793; doi:10.1186/s13104-023-06359-2)
Supplement: Supplementary file 2 — Supplementary Material 2 [file 13104_2023_6359_MOESM2_ESM.docx]

**Data collection pro forma**

**Profile and outcome of patients with Warfarin Toxicity admitted at the National Referral Hospital, Thimphu**

| 1 | Sl no |  |
| --- | --- | --- |
| 2 | CID |  |
| 3 | Hospital registration number |  |
|  |  |  |
|  | **PATIENT PROFILE** |  |
| 4 | Age | _______ years |
| 5 | Sex | ⬜ Male  ⬜ Female |
| 6 | Residence | ⬜ Rural  ⬜ Urban |
| 7 | Level of education | ⬜ Cannot read and write  ⬜ Monastic education / Non-formal education  ⬜ Class PP – VIII  ⬜ Class IX – XII  ⬜ Diploma / Undergraduate  Masters or above |
| 8 | Admission from | ⬜ Emergency Department  ⬜ Outpatient  ⬜ Referral |
|  |  |  |
|  | **WARFARIN DETAILS** |  |
| 9 | Indication for Warfarin | ⬜ Mechanical heart valves  ⬜ Atrial fibrillation  ⬜ Deep vein thrombosis  ⬜ Pulmonary thromboembolism  ⬜ Others |
| 10 | Duration of warfarin therapy | ________ weeks/months/years |
| 11 | Dose of Warfarin (dose that patient had actually taken) | ________ mg |
| 12 | Warfarin dose given to the patient by | ⬜ Self  ⬜ Care giver |
| 13 | Weekly cumulative dose of Warfarin (week prior to event) | ___________ mg |
| 14 | INR prior to admission | ___________ |
| 15 | First INR after admission | ___________ |
| 16 | Presumed cause of warfarin toxicity (as mentioned in patient files) | ⬜ Dosing error – patient  ⬜ Dosing error – physician  ⬜ Dosing error – pharmacist  ⬜ Dosing error – unknown agent  ⬜ Drug interactions  ⬜ Acute illness  ⬜ Liver disease  ⬜ Intentional self-harm  ⬜ Dietary interaction  ⬜ Other causes  ⬜ Cause not identified |
|  |  |  |

|  | **CO-MORBID CONDITIONS** |  |
| --- | --- | --- |
| 17 | Chronic heart failure | ⬜ Yes ⬜ No |
| 18 | Treatment for hypertension | ⬜ Yes ⬜ No |
| 19 | Diabetes | ⬜ Yes ⬜ No |
| 20 | History of ischemic stroke | ⬜ Yes ⬜ No |
| 21 | Coronary artery disease | ⬜ Yes ⬜ No |
| 22 | History of myocardial infarction | ⬜ Yes ⬜ No |
| 23 | Peripheral artery disease | ⬜ Yes ⬜ No |
| 24 | Congestive heart failure | ⬜ Yes ⬜ No |
| 26 | Pulmonary disease | ⬜ Yes ⬜ No |
| 26 | Hepatic disease | ⬜ Yes ⬜ No |
| 27 | Renal disease | ⬜ Yes ⬜ No |
| 28 | Psychiatric disorder | ⬜ Yes ⬜ No |
| 29 | CHA_2_DS_2_-VASc score | _______ |
| 30 | SAMe-TT_2_R_2_ | _______ |
|  |  |  |
| 31 | Serum creatinine | _______ mg/dL |
| 32 | eGFR (by CKD-EPI) | _______ mL/min/1.73 m^2^ |
| 33 | History of malignancy | ⬜ Yes ⬜ No |
| 34 | Active malignancy | ⬜ Yes ⬜ No |
| 35 | Alcohol abuse | ⬜ Yes ⬜ No |
| 36 | Active smoking | ⬜ Yes ⬜ No |
| 37 | Recent bleed | ⬜ Yes ⬜ No |
|  |  |  |
|  | **Concomitant pharmacy** |  |
| 38 | Antiarrhythmics | ⬜ Yes ⬜ No |
| 39 | Atorvastatin | ⬜ Yes ⬜ No |
| 40 | Low dose aspirin (<100 mg) | ⬜ Yes ⬜ No |
| 41 | High dose aspirin (≥100 mg) | ⬜ Yes ⬜ No |
| 42 | Clopidogrel | ⬜ Yes ⬜ No |
| 43 | Antidiabetics | ⬜ Yes ⬜ No |
| 44 | Antihypertensives | ⬜ Yes ⬜ No |
| 45 | Antibiotic | ⬜ Yes ⬜ No |
| 46 | Antifungal | ⬜ Yes ⬜ No |
| 47 | Antiepileptic drug | ⬜ Yes ⬜ No |
|  |  |  |
|  | **WARFARIN TOXICITY** |  |
| 48 | Types of adverse events | ⬜ Bleeding  ⬜ Skin necrosis  ⬜ Any other ______________ |
| 49 | **Bleeding sites (International Society for Thrombosis and Haemostasis ISTH classification of bleeding severity)** | |
| 49a | Intracranial  *If yes, indicate the severity* | ⬜ Yes ⬜ No |
|  |  | ⬜ ISTH 1  ⬜ ISTH 2  ⬜ ISTH 3  ⬜ ISTH 4 |
| 49b | Oral cavity  *If yes, indicate the severity* | ⬜ Yes ⬜ No |
|  |  | ⬜ ISTH 1  ⬜ ISTH 2  ⬜ ISTH 3  ⬜ ISTH 4 |
| 49c | Epistaxis  *If yes, indicate the severity* | ⬜ Yes ⬜ No |
|  |  | ⬜ ISTH 1  ⬜ ISTH 2  ⬜ ISTH 3  ⬜ ISTH 4 |
| 49d | Gastrointestinal  *If yes, indicate the severity* | ⬜ Yes ⬜ No |
|  |  | ⬜ ISTH 1  ⬜ ISTH 2  ⬜ ISTH 3  ⬜ ISTH 4 |
| 49e | Muscle hematoma  *If yes, indicate the severity* | ⬜ Yes ⬜ No |
|  |  | ⬜ ISTH 1  ⬜ ISTH 2  ⬜ ISTH 3  ⬜ ISTH 4 |
| 49f | Haemarthrosis  *If yes, indicate the severity* | ⬜ Yes ⬜ No |
|  |  | ⬜ ISTH 1  ⬜ ISTH 2  ⬜ ISTH 3  ⬜ ISTH 4 |
| 49g | Any other bleed sites | ⬜ Yes ⬜ No |
|  | *If yes, indicate the severity* | ⬜ ISTH 1  ⬜ ISTH 2  ⬜ ISTH 3  ⬜ ISTH 4 |
|  | **TREATMENT AND OUTCOME** |  |
| 50 | Treatment | ⬜ Stopped Warfarin  ⬜ Reduced dose of Warfarin  ⬜ Vitamin K  ⬜ Fresh frozen plasma  ⬜ Packed red blood cells |
| 51 | Date of admission | _____________ (dd/mm/yyy) |
| 52 | Date of discharge | _____________ (dd/mm/yyy) |
| 53 | ICU admission | Yes ⬜ No |
| 54 | Date of ICU admission | _____________ (dd/mm/yyy) |
| 55 | Date of ICU trans out | _____________ (dd/mm/yyy) |
| 56 | Outcome | ⬜ Discharged alive  ⬜ Referred outside  ⬜ Died |

-end of questionnaire-
